# CC360 Pearl: How to Review a Manuscript

**DOI:** 10.1093/crocol/otae042

**Published:** 2024-07-23

**Authors:** Hannah Systrom, Lisa Malter, Hilary K Michel

**Affiliations:** Department of Medicine, Dartmouth Hitchcock Medical Center, Lebanon NH, USA; Department of Medicine, NYU Grossman School of Medicine, New York, NY, USA; Division of Gastroenterology, Nationwide Children’s Hospital, Columbus, OH, USA; Department of Pediatrics, The Ohio State University College of Medicine, Columbus, OH, USA

Engaging with research in any capacity, whether conducting, reviewing, or applying it to practice, is undoubtedly a part of a career in medicine. However, the skills of interpreting and applying research are not always learned as a part of formal residency or fellowship training. Serving as a peer reviewer can be an excellent opportunity to gain exposure to the latest research methods, develop skills in evaluating research quality, provide feedback to strengthen the work, and give back to the research community. ).

In an effort to help improve trainees’ engagement in research and learning in this area, CC360 has developed 2 important initiatives: The CC360 Editorial Fellowship and the Fellow’s Corner section of the Journal.^1,2^ Each Fellow’s Corner includes a “Pearl” that focuses on topics important to trainee career development. This Pearl focuses on how to conduct a high-quality peer review, a skillset that the CC360 Editorial Fellow gains during the fellowship. It also introduces the CC360 Reviewer Guide (Supplementary 1)—a comprehensive resource to summarize best practices.

Learning to review a manuscript is similar to learning endoscopic skills: It is helpful to follow a step-by-step process and refine this process as needed:

1. First-pass review of the paper. It is helpful to start with an initial high-level read of the manuscript to develop a first impression of the research, the main question addressed, and the writing quality.^3^ During this read, take general notes on the themes of the work and questions that arise, and start thinking about how you might summarize the work in one paragraph.
2. Create a summary paragraph of the manuscript. After the first-pass read, draft a 4–5 sentence summary paragraph that encompasses the main research question, methods, how the research is novel, and what is well done. This can be adjusted as you continue to review the manuscript.
3. Review the literature on the topic. If the subject matter is unfamiliar, it is beneficial to review the literature in the area to ensure you understand what has been studied about this subject matter and the most current understanding of the topic. It can also be helpful to review the reference list and use the opportunity to suggest adding citations to the manuscript.^4^
4. Approach each section and start your “major” and “minor” comments. Then, approach the manuscript section by section—abstract, introduction, methods, results, discussion, and tables and figures. Re-read each section and write down “major” and “minor” comments for the editor. Major comments include larger revisions the paper may need—clarifications about the research question, methods, or study population, as well as additional information needed to improve or strengthen the study. Minor comments focus on smaller details such as grammar and formatting. For the abstract, ensure that it is congruent with the manuscript as a whole and provides an “accessible summary” of the paper.^3^ The introduction should lay the foundation of the project by summarizing the work previously done in the field, justifying the need for the work, and clearly stating the study's aim. For the methods, the reader should assess the study population, research, and statistical methods and determine if they are appropriate to answer the research question.^5^ For the results, it is important to understand why the authors reported the results as they did and determine if they answered the study question and are consistent with the discussion. In the review of the discussion, the authors should describe the study findings in the context of prior research^5^ and ensure the manuscripts’ conclusions are evidence-based.^3^ Additionally, all potential limitations should be addressed. The authors should ensure they have outlined the next steps based on the results of their study or how these results impact the field. Lastly, review the tables and figures and ensure they accurately represent the results and discussion sections and add to the reader’s understanding of the work.
5. Consider the impact of the manuscript. While reading and reviewing, it is important to ask these questions: Does the manuscript achieve what it set out to achieve? Does it move the field forward and how? What does it contribute that is unique? Ensure this information is included in your summary paragraph. If the manuscript falls short of these goals, suggest to the authors how they might improve in your major comments.
6. Be constructive, kind, and give back to the community. Remember your job is to be fair, objective, and provide constructive feedback to enhance the manuscript.^5^ Additionally, in serving as a reviewer, you have the ability to make an impact on the current literature, and see your edits reflected in the published work.

After completing the review with a summary paragraph, major and minor comments, reread the manuscript one more time to ensure comments are clear, relevant, and help the author to improve their work. Throughout the review process, reference the CC360 Reviewer Guide (Supplementary 1) which contains an extensive list of resources related to best practices in peer review including ethics, process, and tips.

Using these resources and practicing reviewing manuscripts can help one to hone their review skills and critical thinking. The CC360 Editorial Fellowship enables learning not just about the review process, but also about the subject matter addressed in the manuscripts. Ultimately it is both meaningful and rewarding to see your contributions reflected in the published work and give back to the inflammatory bowel disease research community.

## Supplementary Material

otae042_suppl_Supplementary_Materials
