# Supplementary material for: CC360 Pearl: How to Review a Manuscript
Source: Crohns Colitis 360. 2024 Jul 23;6(3):otae042. doi: 10.1093/crocol/otae042 (PMC11331038; doi:10.1093/crocol/otae042)
Supplement: otae042_suppl_Supplementary_Materials [file otae042_suppl_supplementary_materials.pdf]

733 Third Avenue
Suite 510
New York, NY 10017

212-685-3440
info@crohnscolitisfoundation.org
www.crohnscolitisfoundation.org

CROHN'S & COLITIS FOUNDATION

Crohn's & Colitis Foundation Scientific Journal Reviewer Resource Guide

The Crohn's & Colitis Foundation relies on members of the scientific research community to assess the validity of manuscripts under consideration with our two journals – *Inflammatory Bowel Diseases* and *Crohn's & Colitis 360* – through peer review. Peer review is designed to assess the validity, quality and often the originality of manuscripts for publication. Its ultimate purpose is to maintain the integrity of science by filtering out invalid or poor-quality articles.

The Foundation works to establish and sustain integrity in research publication, including effective peer review and ethical considerations. A vital part of this is ensuring that reviewers and potential reviewers have the best resources and skills to conduct their critiques adequately. The peer review process varies from journal to journal, while this guide provides resources to support researchers and clinicians in becoming more effective and efficient scientific peer reviewers.

Resources for Peer Review

Useful Organizations:

1. **Committee on Publication Ethics (COPE): https://publicationethics.org/**
    - **a.** COPE is a forum for editors of peer-reviewed journals to discuss issues related to the integrity of the scientific record. It supports and encourages editors to report, catalogue and instigate investigations into ethical problems in the publication process. COPE is the recognized global body for establishing journal ethics guidelines and Oxford University Press (OUP; the publisher for both Foundation journals) is a member.
    - **b.** Recommended resources from COPE:
        - i. Ethical guidelines for peer reviewers
        - ii. A short guide to ethical editing for new editors
        - iii. How to handle authorship disputes: a guide for new researchers

2. **International Committee of Medical Journal Editors (ICMJE): https://www.icmje.org/**
    - **a.** ICMJE consists of a group of editors from medical journals who meet annually to discuss the 'Uniform Requirements for Manuscripts Submitted to Biomedical Journals: Writing and Editing for Biomedical Publication'. These guidelines have been extended to cover more than just manuscript preparation, and now include ethical considerations and many editorial issues. All reviewers, editors and authors will benefit from reviewing this valuable and useful resource.
    - **b.** Recommended resources from ICMJE:
        - i. ICMJE | Recommendations | Responsibilities in the Submission and Peer-Review Process
3. **Council of Science Editors (CSE):**
    - **a.** CSE is an international membership organization for editorial professionals publishing in the sciences. CSE's purpose is to serve over 800 members in the scientific, scientific publishing, and information science communities by fostering networking, education,

This guide was developed by Dr. Lisa Malter and Dr. Hilary Michel who serve as Associate Editors for *Crohn's & Colitis 360* and was approved by *Crohn's & Colitis 360* Editor-in-Chief and Deputy Editor, Dr. Miguel Reguerio and Dr. Alan Moss, respectively.

733 Third Avenue
Suite 510
New York, NY 10017

212-685-3440
info@crohnscolitisfoundation.org
www.crohnscolitisfoundation.org

discussion, and exchange. CSE's aim is to be an authoritative resource on current and emerging issues in the communication of scientific information.

   b. Recommended resources from CSE:
      i. Reviewer Roles and Responsibilities

Publishing Groups:

1. OUP's Peer Review Guidance
2. PLOS Peer Review Center
   a. Recommended resources from PLOS:
      i. 10 Tips for Getting Started as a Peer Reviewer
      ii. You've Been Invited to Review. Now What?
      iii. How to Read a Manuscript as a Peer Reviewer
      iv. How to Write a Peer Review
3. SAGE Publishing How to Conduct a Peer Review Video
4. Wiley How to Perform a Peer Review and Wiley Reviewer Resources

How to become a Foundation Journal Reviewer:

1. Begin by logging into **Editorial Manager (EM),** our submission system. If you are interested in becoming a reviewer for *Inflammatory Bowel Diseases,* see here. If you are interested in becoming a reviewer for *Crohn's & Colitis 360,* see here.
   a. Note: If this is your first time visiting these sites, you will need to register a new account.
2. Once logged in, click on your name in the top right corner of the screen and ensure all the following information are included and up to date:
   a. Email address
   b. Title or Preferred Greeting
   c. Full Name
   d. Institutional Affiliation with a complete address
   e. Classifications. You may include up to five classification keywords indicating your area(s) of expertise or interest. This is extremely important as we use these classifications to appropriately pair manuscripts with peer reviewers.
3. Email the *Crohn's & Colitis 360* 's Managing Editor (cc360.editorialoffice@jjeditorial.com) and/or *Inflammatory Bowel Diseases'* Managing Editor (ibd.editorialoffice@jjeditorial.com) to express your interest, and to confirm completion of the above.

This guide was developed by Dr. Lisa Malter and Dr. Hilary Michel who serve as Associate Editors for *Crohn's & Colitis 360* and was approved by *Crohn's & Colitis 360* Editor-in-Chief and Deputy Editor, Dr. Miguel Reguerio and Dr. Alan Moss, respectively.

733 Third Avenue
Suite 510
New York, NY 10017

212-685-3440
info@crohnscolitisfoundation.org
www.crohnscolitisfoundation.org

CROHN'S & COLITIS FOUNDATION

4. Add the editorial office inbox and the domain name @editorialmanager.com to your email's trusted senders. This will ensure that you are receiving system emails and notifications of new invitations.

5. Please refer to this user guide to Editorial Manager for key tasks for reviewers in EM.

733 Third Avenue
Suite 510
New York, NY 10017

212-685-3440
info@crohnscolitisfoundation.org
www.crohnscolitisfoundation.org

CROHN'S & COLITIS FOUNDATION

References

1. What is peer review? Wiley Author Services. Accessed March 23, 2023. https://authorservices.wiley.com/Reviewers/journal-reviewers/what-is-peer-review/index.html
2. COPE Ethical guidelines for peer reviewers — English. Committee on Publication Ethics (COPE) Council. September 2017. Accessed March 23, 2023. https://doi.org/10.24318/cope.2019.1.9
3. COPE Guidelines: A Short Guide to Ethical Editing for New Editors. Committee on Publication Ethics (COPE) Council. May 2019. Accessed March 23, 2023. https://doi.org/10.24318/cope.2019.1.8
4. COPE Guidelines: How to handle authorship disputes: a guide for new researchers. Committee on Publication Ethics (COPE) Council. 2018. Accessed March 23, 2023. https://doi.org/10.24318/cope.2018.1.1
5. ICMJE Recommendations ("The Uniform Requirements"). International Committee of Medical Journal Editors. 2018. Accessed March 23, 2023. https://www.icmje.org/about-icmje/faqs/icmje-recommendations/
6. Responsibilities in the Submission and Peer-Review Process. International Committee of Medical Journal Editors. 2018. Accessed March 23, 2023. https://www.icmje.org/recommendations/browse/roles-and-responsibilities/responsibilities-in-the-submission-and-peer-peview-process.html/
7. Anna Trudgett et al. Reviewer Roles and Responsibilities. Council of Science Editors. March 13, 2020. Accessed March 23, 2023. https://cse.memberclicks.net/2-3-reviewer-roles-and-responsibilities/
8. Ethics. Oxford Academics. Accessed March 23, 2023. https://academic.oup.com/pages/authoring/journals/preparing_your_manuscript/ethics#peer/
9. 10 Tips for Getting Started as a Peer Reviewer. Public Library of Science. Accessed March 23, 2023. https://plos.org/resource/10-tips-for-getting-started-as-a-peer-reviewer/
10. You've Been Invited to Review. Now What? Public Library of Science. Accessed March 23, 2023. https://plos.org/resource/youve-been-invited-to-review-now-what/
11. How to Read a Manuscript as a Peer Reviewer. Public Library of Science. Accessed March 23, 2023. https://plos.org/resource/how-to-read-a-manuscript-as-a-peer-reviewer/
12. How to Write a Peer Review. Public Library of Science. Accessed March 23, 2023. https://plos.org/resource/how-to-write-a-peer-review/
13. SAGE Publishing. How to Conduct a Peer Review. https://www.youtube.com/watch?v=qLONnz4AzsY/ Date published January 5, 2022. Accessed March 23, 2023
14. How to perform a peer review. Wiley Author Services. Accessed March 23, 2023. https://authorservices.wiley.com/Reviewers/journal-reviewers/how-to-perform-a-peer-review/index.html
15. Peer Review Resources. Wiley Author Services. Accessed March 23, 2023. https://authorservices.wiley.com/Reviewers/journal-reviewers/tools-and-resources/index.html/

This guide was developed by Dr. Lisa Malter and Dr. Hilary Michel who serve as Associate Editors for *Crohn's & Colitis 360* and was approved by *Crohn's & Colitis 360* Editor-in-Chief and Deputy Editor, Dr. Miguel Reguerio and Dr. Alan Moss, respectively.
